# Barriers and facilitators to vaccination in Latin America: a thematic synthesis of qualitative studies

**DOI:** 10.1590/0102-311XEN165023

**Published:** 2024-06-21

**Authors:** Javier Roberti, Natalí Ini, Maria Belizan, Juan Pedro Alonso

**Affiliations:** 1 Instituto de Efectividad Clínica y Sanitaria, Buenos Aires, Argentina.; 2 Consejo Nacional de Investigaciones Científicas y Técnicas, Buenos Aires, Argentina.

**Keywords:** Vaccination Hesitancy, Vaccination, Qualitative Research, Review, Vacilación a la Vacunación, Vacunación, Investigación Cualitativa, Revisión, Hesitação Vacinal, Vacinação, Pesquisa Qualitativa, Revisão

## Abstract

Vaccines are often undervalued or underused for a variety of reasons, and vaccine hesitancy is a global challenge that threatens vaccine acceptance and the goals of immunization programs. This review aimed to describe the barriers and facilitators to vaccination in Latin America. The study design was a systematic review and thematic synthesis of qualitative studies reporting on the knowledge or attitudes of adults, parents of children at vaccination age, adolescents and health professionals towards vaccination in Latin America. The databases searched were PubMed, CENTRAL, Scopus, LILACS, SciELO, and CINAHL. A total of 56 studies were included. Facilitators included vaccination being recognized as an effective strategy for preventing infectious diseases and as a requirement for access to social assistance programs, schooling or employment. Recommendations from health professionals and positive experiences with health services were also identified as facilitators. The main barriers were lack of information or counseling, structural problems such as shortages of vaccines and limited hours of operation, the inability to afford over-the-counter vaccines or transportation to health facilities, certain religious beliefs, misconceptions and safety concerns. Qualitative research can contribute to understanding perceptions and decision-making about vaccination and to designing policies and interventions to increase coverage.

## Resumen

Las vacunas suelen estar subvaloradas o desaprovechadas por diversas razones. La vacilación ante las vacunas es un desafío global y representa una amenaza para la aceptación de las vacunas y para los objetivos de los programas de inmunización. El objetivo de esta revisión es describir las barreras y los facilitadores de la vacunación en América Latina. El diseño del estudio fue una revisión sistemática y una síntesis temática de estudios cualitativos sobre conocimientos o actitudes de adultos, padres de niños en edad de vacunación, adolescentes y profesionales de la salud sobre la vacunación en América Latina. Las bases de datos analizadas fueron PubMed, CENTRAL, Scopus, LILACS, SciELO y CINAHL. Se incluyeron 56 estudios. Los facilitadores incluyeron la idea de que la vacunación era reconocida como una estrategia eficaz para prevenir enfermedades infecciosas y un requisito para el acceso a la asistencia social, la escolaridad o el empleo. También se identificaron como factores facilitadores las recomendaciones de los profesionales de la salud y las experiencias positivas con los servicios de salud. Los principales obstáculos fueron la falta de información o asesoramiento, problemas estructurales como la escasez de vacunas y los horarios de funcionamiento limitados, la imposibilidad de pagar vacunas no provistas de forma gratuita o de desplazarse a los centros de salud, ciertas creencias religiosas, conceptos erróneos sobre las vacunas y preocupaciones por la seguridad. La investigación cualitativa puede contribuir a la comprensión de las percepciones y a la toma de decisiones sobre la vacunación y al desarrollo de políticas e intervenciones para aumentar la cobertura de vacunación.

## Resumo

As vacinas são frequentemente subvalorizadas ou subutilizadas por uma série de razões. A hesitação vacinal é um desafio global, sendo uma ameaça à aceitação das vacinas e aos objetivos dos programas de imunização. O objetivo desta revisão é descrever barreiras e facilitadores para a vacinação na América Latina. O desenho do estudo foi uma revisão sistemática e síntese temática de estudos qualitativos sobre conhecimento ou atitudes de adultos, pais de crianças em idade de vacinação, adolescentes e profissionais de saúde sobre vacinação na América Latina. As bases de dados analisadas foram PubMed, CENTRAL, Scopus, LILACS, SciELO e CINAHL. Foram incluídos 56 estudos. Os facilitadores incluíram a ideia de que a vacinação era reconhecida como uma estratégia eficaz para prevenir doenças infecciosas e um requisito para o acesso à assistência social, escolaridade ou emprego. Além disso, recomendações de profissionais de saúde e experiências positivas com serviços de saúde também foram identificados como fatores facilitadores. Os principais obstáculos foram a falta de informação ou aconselhamento, problemas estruturais como escassez de vacinas e horário limitado de funcionamento, incapacidade de comprar vacinas pagas ou se transportar para unidades de saúde, certas crenças religiosas, concepções erradas sobre vacinas e preocupações de segurança. A pesquisa qualitativa pode contribuir para a compreensão das percepções e tomadas de decisão sobre a vacinação e para o desenvolvimento de políticas e intervenções para aumentar a cobertura vacinal.

## Introduction

Vaccination has become a critical tool for reducing the incidence of many infectious diseases and associated mortality ^1^. Its health benefits translate into positive economic outcomes for health systems and society. Indeed, access to vaccines should be an economic development strategy for countries. However, vaccines are often undervalued or underused for a variety of reasons ^1^. Low vaccination coverage remains a public health problem in many regions, including Latin America ^2^ ^,^ ^3^. Achieving effective vaccination coverage depends on several factors, including those related to the quality of health services, individual and community practices, and political decision-making ^4^ ^,^ ^5^. Increasingly higher coverage rates are needed to control target diseases, while both the complexity of vaccine logistics and costs have increased ^6^. Although progress has been made in achieving coverage objectives in Latin America, rates are still below the levels set by the Pan American Health Organization (PAHO), with significant socioeconomic disparities in coverage rates, which vary widely between and within countries ^5^ ^,^ ^6^.

Vaccine refusal is a global challenge, threatening the acceptance of vaccines and the objectives of vaccination programs ^7^ ^,^ ^8^ ^,^ ^9^ ^,^ ^10^. In 2011, the World Health Organization’s (WHO) Strategic Advisory Group of Experts on Immunization (SAGE) established a working group to address “vaccine hesitancy”, defined as a “*delay in accepting vaccination or refusal of vaccines despite the availability of vaccination services*” ^7^ (p. 1). This concept posits a continuum between those who accept all vaccines without hesitation or concern and those who reject them outright, identifying individuals or groups between these two extremes as “hesitant”. More recently, a working group established by the WHO to measure the behavioral and social drivers of vaccination defined vaccine hesitancy as a motivational state of being conflicted or opposed to vaccination ^11^. The concept has become a widely recognized term and has gained traction in vaccine discussions and research over the past decade, particularly in the context of COVID-19 ^12^. However, criticism has been raised, including concerns about the operationalization of vaccine hesitancy in research ^12^ ^,^ ^13^, its oversimplification of vaccine decision-making, and the potential for stigmatization of hesitant individuals ^14^. There are also warnings about the use of frameworks designed for the Global North in the Global South and the importance of considering regional differences in beliefs and behaviors for effective public health research ^15^.

Vaccine hesitancy does not necessarily equate to low coverage, but lack of confidence in vaccination has been linked to low vaccine uptake and a subsequent increase in morbidity and mortality from vaccine-preventable diseases. Inequalities in routine immunization in Latin America have been exacerbated by the effects of the COVID-19 pandemic on vulnerable populations and by misconceptions about vaccines spread by anti-vaccine movements ^16^. To improve vaccination coverage in Latin America, it is important to identify the factors that act as barriers to vaccine uptake. Regarding access to vaccines, the implementation of vaccination policies in the region faces several challenges to resolve inequalities in vaccination coverage, such as lack of adequate financial support and common regulations among countries, problems in the introduction of new vaccines, deficiencies in the training of health personnel and low awareness of vaccine-preventable diseases among the general population ^17^.

Most primary studies and reviews report findings from high-income countries, and research on other regions is needed to provide contextual information on perceptions and attitudes towards vaccination ^18^ ^,^ ^19^. It has been highlighted that some factors underlying vaccine mistrust - such as the appeal of conspiracy theories - can only be described using qualitative methods ^20^. Qualitative studies have also described contextual and complex factors that influence decision-making processes ^21^ ^,^ ^22^ ^,^ ^23^ ^,^ ^24^. This review aimed to describe different barriers and facilitators to vaccination in Latin America by synthetizing qualitative evidence.

## Methods

This systematic review was conducted according to the Enhancing Transparency of Reporting the Synthesis of Qualitative Research (ENTREQ) framework ^25^. Comprehensive search strategies were used to identify all available studies. This work included qualitative studies that used focus group discussions, interviews, direct observation, case studies, ethnography, and action research published in English, Spanish and Portuguese. The study populations were composed of adults, parents (those who raise or care for a child or play the role of guardian), adolescents (10-19 years of age) and health professionals (physicians, nurses, vaccinators). The outcomes assessed were knowledge (information about vaccination) and attitudes (opinions about vaccination involving a related act or its omission). The studies evaluated were conducted in countries in Latin America, including Puerto Rico. Studies conducted in Latino communities in countries outside Latin America and studies that used surveys and questionnaires were excluded.

### Data sources and search

**Figure 1:**
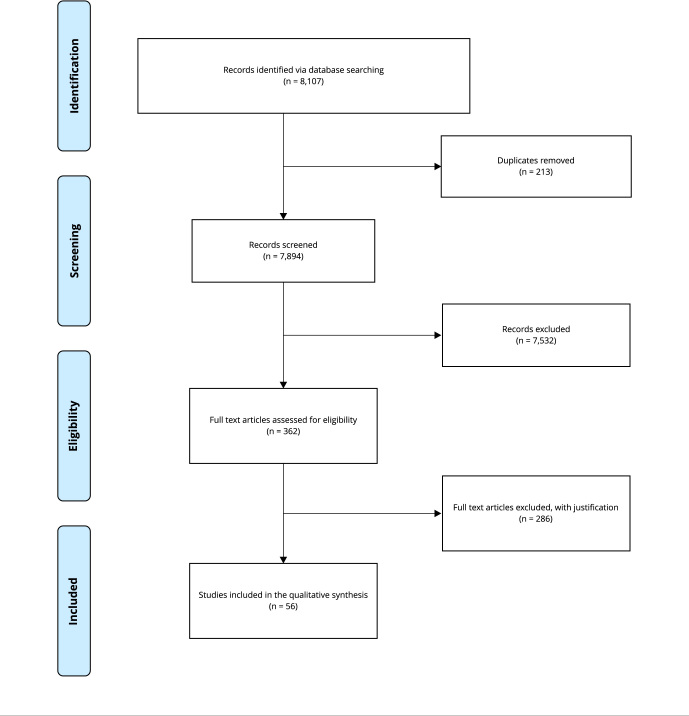
PRISMA (Preferred Reporting Items for Systematic Reviews and Meta-Analyses) ^95^ flowchart.

The following electronic databases were searched: PubMed, CENTRAL, Scopus, LILACS, SciELO, and CINAHL. The electronic search was conducted in January 2022. The terms used were related to knowledge, attitudes and practices regarding vaccination among participants in Latin America. No date restrictions were imposed on the search strategy (Supplementary Material: https://cadernos.ensp.fiocruz.br/static//arquivo/suppl-e00165023_6423.pdf). Two authors (J.R. and N.I.) screened the titles and abstracts of the search records using Covidence (https://www.covidence.org/) and a screening guide. These two authors identified potentially eligible studies and retrieved the full text of selected records. Then, three authors (J.R., N.I., and M.B.) independently screened all full texts for eligibility and resolved discrepancies by discussion and consensus. No studies were excluded based on quality. The following characteristics were extracted: year of publication, country, population, number of participants, data collection, methodology, analysis, and research questions. A PRISMA (Preferred Reporting Items for Systematic Reviews and Meta-Analyses) flowchart with a summary of the records searched and selected was generated (Figure 1).

### Data analysis

Thematic synthesis was used as described by Thomas & Harden ^26^. The text under the results/findings sections of the included studies was imported into Atlas.Ti software (http://atlasti.com/). Two researchers (J.R. and N.I.) performed line-by-line coding, conceptualized the data and inductively identified concepts. To assess coding concordance, reconcile discrepancies and validate codebooks, two researchers independently coded four studies. Inter-coder agreement was assessed using Cohen’s kappa, with a kappa ≥ 0.80 widely accepted as demonstrating high coding concordance and semantic reliability. For subsequent articles, the text was coded into existing concepts, and a new concept was created when needed. Similar concepts were grouped into themes. To ensure that coding captured all relevant issues and reflected the primary data, researcher triangulation was used, guaranteeing that codes captured relevant data. Two researchers (J.P.A. and M.B.) reviewed the preliminary themes and discussed themes with the first two researchers.

## Results

### Characteristics of the included studies

Searches were run conducted in January 2022. It yielded 8,107 results, and after screening titles, abstracts and full texts, 56 articles published from 2007 to January 2022 were included (Figure 1). Box 1 provides a summary description of the included articles. Most studies were based on individual interviews or focus groups. The number of study participants ranged from 7 to 362. The main vaccines discussed by the included studies were the HPV vaccine (n = 15), childhood vaccines (n = 11), and influenza (n = 8) and maternal vaccines (n = 7). Regarding populations included in the studies, the most prominent were parents of children at vaccination age (n = 23), health professionals (n = 11) and pregnant women (n = 9). The two countries with the most studies were Brazil (n = 22) and Peru (n = 13).

**Box 1:** Characteristics of the included studies.

STUDY (YEAR)	COUNTRY	SAMPLE SIZE	PRIMARY FOCUS/OBJECTIVE	DATA COLLECTION METHOD	DATA ANALYSIS
Aragão et al. ^85^ (2019)	Brazil	23	Nurses’ perceptions of vaccination	Interviews	Thematic analysis
Arams et al. ^39^ (2021)	Chile	30	Maternal factors and family dynamics that affect HPV vaccination behavior	Interviews	Grounded theory
Barbieri et al. ^55^ (2015)	Brazil	30	Parents’ decision-making process	Interviews	Content analysis
Barbieri et al. ^43^ (2017)	Brazil	30	Deciding not to vaccinate	Interviews	Content analysis
Barrington et al. ^78^ (2008)	Dominican Republic	25	HIV vaccine and sexual behavior	Interviews	Content analysis
Barrington et al. ^81^ (2007)	Dominican Republic	25	HIV vaccine	Interviews	Content analysis
Bazán et al. ^84^ (2017)	Peru	70	Health professionals’ attitudes and influenza vaccine	Interviews	Thematic analysis
Bingham et al. ^38^ (2009)	Peru	299/50	Sociocultural issues of HPV vaccination	Interviews and focus groups	Table matrix technique
Burghouts et al. ^40^ (2017)	Venezuela	30	Vaccination in infants and children	Interviews	Thematic analysis
Calo et al. ^77^ (2015)	Puerto Rico	23	HPV vaccine and ethnic identity	Focus group	Thematic analysis
Carcelen et al. ^54^ (2021)	Peru	12	Pregnant women’s perceptions and attitudes towards vaccination	Interviews	Theoretical framework
Castillo-Neyra et al. ^71^ (2020)	Peru	70	Rabies vaccines during the pandemic	Focus group	Thematic analysis
Clavé Llavall et al. ^58^ (2021)	Peru	21	Vaccination uptake in nurses and teachers	Interviews	Theory informed thematic analysis
Clavijo et al. ^79^ (2016)	Peru	32	Perception of rabies vaccines	Interviews and observation	Thematic analysis
Cordoba-Sanchez et al. ^37^ (2019)	Colombia	110	Barriers and facilitators for HPV vaccination in school children	Interviews and focus groups	Content analysis
Costa e Silva et al. ^73^ (2013)	Brazil	30	Social representations of the influenza vaccine	Interviews	Thematic analysis
Couto & Barbieri ^42^ (2015)	Brazil	15	Vaccine refusal in high-income families	Interviews	Content analysis
Duarte et al. ^75^ (2019)	Brazil	74	Feelings and perceptions regarding vaccination in primary health care	Interviews	Thematic content analysis
Duarte et al. ^47^ (2020)	Brazil	74	Vaccination as a scheduled demand and access	Interviews	Thematic content analysis
Duarte et al. ^56^ (2021)	Brazil	74	Access to vaccination and its organizational aspects in primary health care	Interviews	Thematic content analysis
Escobar-Díaz et al. ^70^ (2017)	Colombia	36	Reasons for refusing vaccination	Interviews and focus groups	Thematic analysis
Fernández et al. ^67^ (2014)	Puerto Rico	30	Perception of the HPV vaccine	Focus group	Grounded theory
Figueiredo et al. ^41^ (2011)	Brazil	22	Experiences of immunization in children	Interviews	Thematic analysis
Fleming et al. ^57^ (2018)	El Salvador	326	Maternal immunization against influenza	Interviews and focus groups	Thematic analysis
Galea et al. ^82^ (2017)	Peru	36	Knowledge and acceptance of the HPV vaccine	Interviews and focus groups	Content analysis
Garcia et al. ^59^ (2020)	Peru	66	Knowledge of influenza and immunization	Interviews and focus groups	Thematic analysis
Gonçalves & Machado ^36^ (2008)	Brazil	16	Immunization in carers	Interviews	Content analysis
González-Block et al. ^60^ (2021)	Brazil, Chile, Paraguay, Peru, and Uruguay	150	Confidence, complacency and convenience of influenza vaccination	Focus group	Content analysis
González-Block et al. ^7^ (2021)	Peru	28	Confidence in the influenza vaccine	Focus group	Qualitative analysis
Handy et al. ^50^ (2017)	Dominican Republic	96	Access to information on vaccination	Focus group	Deductive approach based on conceptual framework
Islam et al. ^61^ (2018)	Argentina	23	Mothers’ rationale regarding their preferences for HPV vaccination	Focus group	Thematic analysis
Liebermann et al. ^35^ (2020)	Dominican Republic	64	Barriers and facilitators for HPV vaccination	Focus group	Content analysis
Malik et al. ^72^ (2021)	Argentina, Brazil, Honduras, Mexico, and Peru	162	Pregnancy and immunization	Interviews and focus groups	Thematic analysis
Malik et al. ^62^ (2021)	Argentina, Brazil, Honduras, Mexico, and Peru	33	Health care providers	Interviews	Thematic analysis
Marbán-Castro et al. ^49^ (2020)	Colombia	30	Acceptance of a hypothetical vaccine against Zika	Interviews	Grounded theory
Melo et al. ^34^ (2013)	Brazil	14	Adolescents’ perception of vaccination	Interviews	Content analysis
Morais & Quirino ^33^ (2010)	Brazil	7	Immunization and premature babies	Interviews	Content analysis
Muyulema et al. ^63^ (2020)	Ecuador	26	Mothers’ knowledge of vaccination	Interviews	Thematic analysis
Oliveira et al. ^32^ (2019)	Brazil	17	Nurses’ knowledge of vaccination	Interviews	Phenomenological analysis
Oliveira et al. ^66^ (2018)	Brazil	124	Influenza vaccine for workers	Experience-based narratives	NA
Pereira et al. ^31^ (2013)	Brazil	10	Pregnancy and refusal of the H1N1 vaccine	Interviews	Content analysis
Pérez-Guerra et al. ^45^ (2012)	Puerto Rico	70	Interests in participation in dengue vaccine trials	Interviews and focus groups	Thematic analysis
Petrocy & Katz ^83^ (2014)	Guatemala	40	Attitudes and beliefs regarding cervical cancer and the HPV vaccine	Interviews	Content analysis
Piñeros et al. ^30^ (2010)	Colombia	14	Introducing the HPV vaccine	Interviews	Content analysis
Rees et al. ^53^ (2017)	Nicaragua	20	HPV vaccine	Interviews	Deductive approach
Ropero Alvarez et al. ^64^ (2021)	Argentina, Brazil, Honduras, Mexico, and Peru	252	Maternal and neonatal immunization in Latin America	Interviews and focus groups	Table matrix technique
Santana et al. ^29^ (2019)	Cuba	72	Knowledge, beliefs and meanings of preventive vaccines	Interviews	Deductive approach
Sealy et al. ^80^ (2021)	Trinidad and Tobago	33	Barriers and facilitators for potential acceptance of the HPV vaccine by mothers of female adolescents	Interviews	Thematic analysis
Sherlock et al. ^28^ (2013)	Brazil	16	Mothers’ perception of HIV vaccination for infants	Interviews	NA
Silva et al. ^74^ (2021)	Brazil	10	Feelings of preadolescents and adolescents towards HPV vaccination	Interviews	Content analysis
Silva et al. ^68^ (2018)	Brazil	55	Knowledge of HPV vaccination	Interviews	Collective subject discourse
Simas et al. ^52^ (2021)	Mexico	54	Experiences with maternal vaccination	Interviews and focus groups	Thematic analysis
Simas et al. ^8^ (2021)	Panama	56	Pregnant women’s views and attitudes towards maternal immunization	Interviews and focus groups	Deductive-inductive approach
Simas et al. ^44^ (2021)	Brazil	60	Barriers and facilitators for maternal immunization	Interviews and focus groups	Deductive-inductive approach
Véliz et al. ^51^ (2016)	Chile	102	Parents’ knowledge and attitudes towards vaccination	Open questions	Content analysis
Wiesner et al. ^69^ (2010)	Colombia	196	Parents’ acceptance of HPV vaccination	Focus group	Content analysis

NA: not available.

### Facilitators

#### The perceived benefit of vaccines

Vaccination was widely recognized as an effective strategy for preventing contagious diseases at the individual and collective levels ^8^ ^,^ ^27^ ^,^ ^28^ ^,^ ^29^ ^,^ ^30^ ^,^ ^31^ ^,^ ^32^ ^,^ ^33^ ^,^ ^34^ ^,^ ^35^ ^,^ ^36^ ^,^ ^37^ ^,^ ^38^ ^,^ ^39^ ^,^ ^40^ ^,^ ^41^ ^,^ ^42^ ^,^ ^43^ ^,^ ^44^ ^,^ ^45^, and several participants reported perceiving vaccination as important to public health because it protected children and helped them avoid disease later in life ^8^ ^,^ ^29^ ^,^ ^30^ ^,^ ^36^ ^,^ ^37^ ^,^ ^38^ ^,^ ^39^ ^,^ ^40^ ^,^ ^41^ ^,^ ^42^ ^,^ ^43^ ^,^ ^44^ ^,^ ^46^. Some participants stated that vaccines could cure diseases ^38^. Vaccination were described as an expression of good parenting ^42^, a value ^43^ ^,^ ^44^ and a requirement for access to important benefits, such as admission to school ^35^ ^,^ ^45^ or the labor market ^47^ and inclusion in social welfare programs ^48^. The decision to vaccinate was seen as an act of love, a responsibility ^8^ ^,^ ^43^ ^,^ ^44^. Brazilian mothers believed that the immune system of premature babies was weaker and more susceptible to infection ^33^.

“*We have to prevent diseases; we have to get vaccinated. They say that there was no immunisation in the past, and people got childhood paralysis, problems with the legs, arms. Nowadays, vaccines are here to prevent these kinds of things. There is yellow fever, flu*” (Brazilian mother) ^41^ (p. 601).

#### Acceptance of health care provider recommendations

Although many participants expressed concerns about the safety of vaccines, recommendations from health care providers were generally accepted ^8^ ^,^ ^49^ ^,^ ^50^ ^,^ ^51^ ^,^ ^52^. Almost all participants reported seeking health information from hospitals or health center campaigns ^8^ ^,^ ^29^ ^,^ ^36^ ^,^ ^44^ ^,^ ^53^, and women were accustomed to requesting additional information and searching for evidence on the internet ^8^ ^,^ ^31^ ^,^ ^39^ ^,^ ^54^ ^,^ ^55^. In Brazil, vaccination cards used by parents included basic information on vaccines and schedules ^41^.

“*I get vaccinated with all the* [vaccines] *the doctor tells me because she knows more and says it for the wellness of you and your baby. Me,* [the recommendations] *I mostly follow is what the doctors tell me*” (pregnant woman from Peru) ^54^ (p. 830).

#### Positive experiences and support from significant others

Peer reports, positive experiences of other pregnant women who had been vaccinated ^8^ and simply following the family tradition of vaccination were the driving forces behind participants’ decisions to get vaccinated ^43^. In addition, participants highlighted the positive aspects and convenience of using the vaccination card ^41^ and their satisfaction with the condition of health facilities or vaccination centers ^56^ as reasons for adherence to schedules.

“*Hygiene is a very serious thing, so you have to check these details, because this gives you confidence. So, if you see a disorganised and dirty place can be off-putting, you won’t take your child there. I usually ask to see the expiration date of the vaccines*” (Brazilian woman) ^56^ (p. 6).

Pregnant women identified themselves as the main decision-makers in terms of vaccination, but discussed the matter with their partners, mothers, other family members, female friends and health professionals ^8^ ^,^ ^40^ ^,^ ^44^ ^,^ ^54^ ^,^ ^57^. For the HPV vaccine, both parents were often involved in the decision to vaccinate ^27^ ^,^ ^37^ ^,^ ^38^, based on trust in the health system and discussion of sexual practices ^39^.

### Barriers

#### Lack of information

Participants highlighted aspects related to the lack of guidance on vaccination and post-vaccination, revealing situations in which professionals’ knowledge was below the desired level to provide guidance ^28^ ^,^ ^34^ ^,^ ^37^ ^,^ ^47^ ^,^ ^50^ ^,^ ^51^ ^,^ ^52^ ^,^ ^56^ ^,^ ^58^ ^,^ ^59^ ^,^ ^60^ ^,^ ^61^ ^,^ ^62^ ^,^ ^63^ ^,^ ^64^, in which vaccines were administered without discussion between health professionals and individuals ^40^, and in which misinformation was widely disseminated ^35^ ^,^ ^37^ ^,^ ^52^ ^,^ ^54^ ^,^ ^65^. In particular, participants often underestimated the prevalence of the target disease ^28^ ^,^ ^66^ and lacked knowledge about HPV and the vaccine ^37^ ^,^ ^58^ ^,^ ^67^ ^,^ ^68^ ^,^ ^69^. Most mothers were unaware of the difference between special vaccines and those available to the general population, also revealing limited knowledge of basic pediatric vaccination ^28^.

“*Usually we arrive and are already vaccinated, but nothing is said about the vaccine or about the benefits and pros of the vaccine. Usually, we’ll be vaccinated more by the sense of obligation, sometimes we’re not really aware of the benefits, are we?* [...] *Look, it was not a service with clarification. The person only takes you to the room where the vaccination will be done and ready, without any kind of guidance*” (Brazilian user) ^47^ (p. 4).

#### Safety concerns

Safety concerns were very common among pregnant women in relation to their pregnancy and the health of their babies, as participants feared that they could cause deformities and disabilities ^52^ ^,^ ^54^ ^,^ ^55^ ^,^ ^59^ ^,^ ^70^ and perceived potential side effects, mostly related to fever, local pain ^37^ ^,^ ^55^ ^,^ ^59^ ^,^ ^71^, diarrhea, flu, vomiting, delay in the appearance of teeth, headache and abdominal pain ^40^. They believed that the target condition was controlled or not severe enough and that the vaccine was not effective or safe ^42^ ^,^ ^54^ ^,^ ^57^ ^,^ ^59^ ^,^ ^60^. Participants were concerned about potentially harmful components such as mercury ^8^ ^,^ ^51^. Side effects were perceived as a result of the booster doses of some vaccines ^63^. Safety concerns about the influenza vaccine, even over minor events, were common, especially among pregnant women ^7^ ^,^ ^31^ ^,^ ^57^ ^,^ ^59^ ^,^ ^72^ ^,^ ^73^. Another concern that acted as a barrier to vaccination was the fear of needles ^74^ ^,^ ^75^. For the HPV vaccine, concerns included the lack of long-term studies and side effects such as those observed in Carmen de Bolivar, Colombia (in 2014, hundreds of girls in the town reported various mysterious symptoms after receiving the HPV vaccine ^76^) ^35^ ^,^ ^37^ ^,^ ^67^ ^,^ ^69^. Participants were also concerned about the safety and effectiveness of new or experimental vaccines, such as the HIV vaccine ^77^ ^,^ ^78^ and the dengue and Zika vaccines ^45^ ^,^ ^49^. They also complained about the economic interests of pharmaceutical companies that could drive vaccination programs ^30^ ^,^ ^42^ ^,^ ^60^.

“*If children are vaccinated, they immediately get a fever, so they have to give us medication for these diseases. Because of the fever, the child gets hot from the inside, in its belly, and therefore they also vomit and get diarrhoea. Health professionals who vaccinate children do not give us medicine for the diseases caused by these vaccines, and if I go to a medical centre, they can’t help me either. That’s when I get mad*” (Venezuelan mother, Warao Amerindian community) ^40^ (p. 8).

#### Religious and cultural beliefs

Religion can also act as a barrier to some vaccines, particularly in rural areas or among indigenous populations, because of beliefs that health is related to God’s will, that the target disease is caused by supernatural forces that cannot be affected by a vaccine, or that if one’s ancestors survived without a vaccine, it is unnecessary now ^8^ ^,^ ^28^ ^,^ ^35^ ^,^ ^38^ ^,^ ^69^ ^,^ ^73^ ^,^ ^79^ ^,^ ^80^. Indigenous groups perceived that vaccination had a hidden objective of harming them by causing disease and/or sterilizing them, benefiting extractive industries in retaliation for protests ^40^ ^,^ ^79^, or that the vaccine was the mark of the devil ^79^. Some participants chose not to get vaccinated because they supported values related to a more natural lifestyle, such as less medical intervention ^43^ ^,^ ^55^. In Paraguay, the idea that the vaccine could cause the disease was widespread ^32^ ^,^ ^62^.

“*Now there are rumours that vaccines are bad, that they bring diseases and that they contain a poison that with time will kill you so that the indigenous people will be exterminated, also because of the number 666, that is why now I am afraid of vaccines. I used to believe that it was effective but with these comments I no longer have confidence*” (Peruvian user, Awajú community) ^79^ (p. 214).

#### Concerns about vaccination schedules

In relation to the influenza vaccine, although older adults and parents of children showed a clear preference for vaccination, some participants questioned the need for annual vaccination and its effectiveness in some at-risk groups ^7^ ^,^ ^59^. In fact, the influenza vaccine was sometimes perceived as having a calendar similar to other vaccines that are administered once or a few times in a person’s lifetime ^59^. Brazilian participants criticized the schedule and the inefficient use of the vaccination card ^42^ ^,^ ^47^. The schedule was also criticized in relation to the early age at which infants receive vaccines and the high number of vaccines included ^42^. The individualization of the child’s vaccination in relation to the vaccination calendar was a variation of behavior within the cultural norm of vaccination ^43^ ^,^ ^55^.

“*I think the vaccine schedule in Brazil is an exaggeration. And in these first years of a child’s life, when the body is so small, I don’t know... It’s a lot of poison for such a little body, in such a short time, so concentrated. So, the idea was never to give the complete schedule*” (Brazilian mother) ^42^ (p. 112).

#### Misconceptions and stigma

A common barrier to vaccination against sexually transmitted diseases or diseases perceived to be associated with sexual behavior is stigma ^28^ ^,^ ^30^ ^,^ ^53^ ^,^ ^78^ ^,^ ^81^. Cervical cancer was associated with isolation resulting from beliefs about its association with HPV and about it being a sign of sexual promiscuity ^53^. Therefore, vaccination against HPV was perceived as a sign of promiscuous sexual behavior. Misconceptions about HPV and the vaccine are related to safety and a concern that the vaccine would enable sexual relations ^30^ ^,^ ^37^ ^,^ ^39^ ^,^ ^53^ ^,^ ^67^ ^,^ ^68^ ^,^ ^69^ ^,^ ^80^ ^,^ ^82^ ^,^ ^83^.

“*I wouldn’t give my daughter the* [HPV] *vaccine because in a way it’s telling her to have sex and do what you want*” (Colombian parent) ^69^ (p. 966).

#### Structural problems in health facilities and the health system

From the perspective of users, the main barriers to vaccination were sporadic vaccine shortages at the health facility level ^8^ ^,^ ^35^ ^,^ ^52^ ^,^ ^62^ ^,^ ^64^ ^,^ ^75^, long waiting times ^28^ ^,^ ^64^ ^,^ ^66^ ^,^ ^67^ ^,^ ^72^ and limited working hours, especially for those who could not or did not want to take time off work to get vaccinated ^7^ ^,^ ^8^ ^,^ ^28^ ^,^ ^47^ ^,^ ^53^ ^,^ ^56^ ^,^ ^66^ ^,^ ^70^ ^,^ ^71^. Vaccine shortages particularly affected primary care centers and rural and suburban areas. Vaccine supply challenges often resulted from procurement mechanisms, disparities in procurement responsibilities and infrequent delivery schedules. Concerns about the way people were treated by health care providers ^52^ ^,^ ^63^ ^,^ ^64^ ^,^ ^75^, low quality of care in the public system ^52^ ^,^ ^64^ - in relation to aspects such as the ability of vaccinators to interact effectively with girls in the case of HPV vaccine, for example -, injection safety practices and the quality of services at health facilities ^38^ ^,^ ^66^ ^,^ ^71^ ^,^ ^72^ were also reported. Geographical accessibility and adverse climatic conditions that affected travel to health facilities, especially in rural areas, remained a significant barrier ^7^ ^,^ ^28^ ^,^ ^47^ ^,^ ^53^ ^,^ ^56^ ^,^ ^66^ ^,^ ^70^. Health care professionals reported problems reaching certain groups, such as migrants and children who dropped out of school ^58^ ^,^ ^62^ ^,^ ^70^.

“*It happened to me that there were about two hundred patients in a line in front of me and there was only one-person vaccinating*” (Argentine user) ^72^ (p. 4).

In the case of maternal care, some participants switched between the private and public systems; and some physicians in the private sector did not fully follow recommendations for maternal immunization or referred women to the public system to access free vaccination ^44^ ^,^ ^62^.

“*Many women have their prenatal control at private facilities, and they come late* [to the vaccination room]*, when they already missed the deadline to Tdap, and then sometimes they are not protected against tetanus. They did not get the influenza vaccine. The hepatitis scheme is not appropriate. Every ten pregnant women who are coming to us from the private sector, eight do not have the proper vaccine scheme, because they were not referred here at the right time to get these vaccines in a timely manner*” (Brazilian nurse) ^62^ (p. 3).

#### Financial problems

Limited availability of human and material resources, lack of funding for outreach activities, high turnover of health workers and low salaries were barriers mentioned by professionals ^58^ ^,^ ^62^ ^,^ ^70^. Another significant barrier related to the structural barriers described above was the fact that if a vaccine was not available at a public vaccination center, people could not always afford it ^58^ ^,^ ^60^ ^,^ ^67^ ^,^ ^75^. To avoid out-of-pocket expenses for vaccines, people were directed to the public system ^8^ ^,^ ^75^. In addition, there were times when people could not afford transportation to the health facilities where they were to receive vaccines ^58^ ^,^ ^60^ ^,^ ^67^ ^,^ ^75^.

“...*the vaccines that are paid, these my boys did not take no, because I cannot afford to pay. As for the meningitis vaccine, I, for example, did not vaccinate them, because it was expensive for me to pay. These payments I wanted to vaccinate my children, but I could not*” (Colombian parent) ^75^ (p. 4).

#### Insufficient training of health professionals

Health professionals were vaccinated for their own protection and that of their families and patients ^84^. Health care providers reported inadequate training in immunization, including for childhood vaccines. Vaccination service professionals were unaware of vaccination coverage and drop-out rates in the territory ^62^ ^,^ ^85^. Health authorities praised the scientific events sponsored by the vaccine industry and found the information provided by manufacturers very useful ^30^.

“*I would have liked more, in fact today we were talking with my colleagues about the issue of saying for example where we could do a course, a course, in which they give us a certificate that we did the course about vaccines*” (Argentine nurse) ^62^ (p. 3).

#### Social violence

In El Salvador, Honduras, Ecuador and Colombia, fear of crime and social violence by gang members was cited as a barrier to vaccination, as limited access to health facilities undermined outreach efforts ^57^ ^,^ ^64^ ^,^ ^70^.

“*In this area, there are already hot zones, such as the northern zone, where there is a displaced population and conflicts have arisen. During the last vaccination campaign, we were unable to proceed due to an armed strike. It is challenging to send a team as they are at risk of being robbed*” (Colombian professional) ^70^ (p. 3).

## Discussion

Among the facilitators, vaccination was recognized as an effective strategy to prevent infectious diseases, but also as a prerequisite for access to important benefits such as social assistance, schooling or a job. Recommendations from health professionals and positive experiences with health services were also identified as facilitators. The main barriers to vaccination were lack of information or advice, structural problems such as shortages of vaccines and limited opening hours of health facilities, inability to afford over-the-counter vaccines or transportation to health facilities, certain religious beliefs, misconceptions and safety concerns. Our findings highlight the multifaceted nature of vaccine acceptance and hesitancy, shedding light on factors at different levels. The identification of facilitators underscores the role of informed decision-making and trusted sources of information, while the barriers emphasize the challenges that need to be addressed to ensure equitable vaccine access and uptake.

Following the initial search for this review, additional studies on the COVID-19 vaccine were identified, enriching our findings. In Grenada, trust in medical advice, vaccine efficacy and travel and social activity mandates facilitated vaccination, while barriers included the perceived low threat of COVID-19, preference for natural remedies and concerns about accessibility and misinformation ^86^. In Argentina, vaccine acceptance was high, driven by social responsibility arguments, but doubts about safety and adverse effects persisted, particularly among educated mothers ^87^. In Peru, vaccine efficacy, long-term health effects and government information influenced vaccination decisions, with some people perceiving promotional strategies as a violation of human rights ^88^. Brazilian nurses identified both strengths, such as teamwork and innovative interventions, and weaknesses, such as lack of training and communication, in the immunization campaign. In addition, reports of childhood vaccine hesitancy revealed fears about the experimental status of vaccines, misinformation and the key role of health professionals in rebuilding trust ^89^. For the first time, public media disseminated misinformation and discouraged vaccination, which was linked to the lack of government coordination in vaccination efforts, a significant contributor to vaccine hesitancy ^89^. Discursive conflicts surrounding COVID-19, including some that minimized the severity of the disease, led to an underestimation of the importance of achieving collective immunity via vaccination ^89^. Among indigenous populations in Guatemala, barriers to vaccination included a lack of culturally sensitive information, misinformation due to mistrust of the government and access issues ^90^. Religion also played a complex role, potentially facilitating or hindering vaccination efforts ^90^. Based on these findings, it is appropriate to examine the politicization of the vaccine and the impact of this particular vaccine on people’s confidence in other vaccines.

Our findings are consistent with those described in a recent review of barriers in the Latin American region, which also cited issues such as inadequate information from health authorities and lack of awareness of the availability, effectiveness and safety of certain vaccines ^17^. In other low- and middle-income countries, concerns about adverse effects of immunization and mistrust of immunization programs were the most common barriers ^24^. In high-income settings, barriers included misleading knowledge, beliefs and perceptions about vaccines and general negative attitudes towards vaccination ^91^. Several socioeconomic factors affected vaccine uptake in all settings, such as ethnicity and low socioeconomic status ^91^.

Some barriers to vaccination are related to the health system and service delivery. Inability to obtain vaccines at health facilities due to vaccine shortages ^9^ ^,^ ^35^, long waiting times and limited vaccination schedules ^8^ ^,^ ^28^ were barriers identified in this review. This wide range of problems exacerbates existing programmatic and health system challenges to childhood immunization services ^24^, such as lack of resources at health facilities (e.g., lack of human and financial resources for outreach activities). At the policy level, several studies have identified compulsory vaccination as a facilitator of school enrolment and access to key social benefits ^35^ ^,^ ^48^.

Barriers to access to health services that limit the demand for vaccines were also identified, such as geographical barriers and lack of resources to travel to health centers. In some Central American countries, social violence was also mentioned as a problem for access to health facilities ^57^. There are also barriers and facilitators at the social and interpersonal levels. As noted in another review, trust in the recommendations of health care providers is one of the main reasons why vaccine-hesitant parents change their minds, and parents who receive information from physicians are less vaccine-hesitant than those who receive information from other sources ^92^. In fact, decisions are strongly influenced by trust in vaccine information, both in terms of content and source ^92^. Fear of vaccine side effects and distrust of the pharmaceutical industry are also common barriers. Our findings show a lack of information from the community and a need for reliable information ^28^ ^,^ ^34^, which contrasts with the lack of time and training in communication skills on the part of some health professionals ^70^.

At the societal level, stigma was identified as a barrier to HPV vaccination because of the community’s association of HPV vaccine demand with sexual promiscuity or early sexual initiation ^53^. Low risk perception among younger children and parents was a barrier to HPV vaccination. This was particularly evident in populations in which religious beliefs discourage premarital sexual activity. Parents were often concerned about the potential encouragement of sexual activity associated with vaccination and the difficult conversations associated with vaccination decisions due to the inherent link to sexuality. Lack of familiarity with the new HPV vaccine leads to concerns about safety and efficacy ^93^. Strategies to improve vaccination coverage should be sensitive to cultural beliefs, socioeconomic disparities and regional differences. In addition, policy efforts should consider the societal impact of stigma and the potential influence of religious norms on vaccination decisions.

Several studies have identified barriers to migrant vaccination ^62^ ^,^ ^70^, but no studies have examined this issue in depth. Many barriers to migrant vaccination when arriving in a new country arise from a lack of information about vaccination and concerns about side effects, safety and low efficacy ^94^. Engaging with newcomer communities and leaders to better understand their concerns can be beneficial in addressing these concerns and promoting vaccination in ways that are accessible and acceptable to these citizens ^94^.

The review has important limitations. In addition to selection and publication bias, most of the studies identified were concentrated in a few countries, and certain barriers are context-specific. The heterogeneity of methodological designs, quality of reporting, populations and types of vaccines made it difficult to synthesize the findings. In addition, there were very few publications on barriers to vaccination in subpopulations such as adolescents, migrants and indigenous groups. Most studies investigating determinants of HPV vaccination decisions focused on parents of adolescents. Lastly, some studies addressed the experience of vaccination during the COVID-19 pandemic and the implementation of mass vaccination campaigns; however, the impact of these processes on perceptions of vaccination policy in general was not fully known at the time, although the negative impact of the pandemic on attitudes towards vaccination was documented.

To our knowledge, this is the first systematic review of qualitative studies on the determinants of vaccination in Latin America. The main barriers to vaccination were lack of information or advice, shortages of vaccines, limited hours of operation, long distances to health facilities, certain religious beliefs, misconceptions and safety concerns. Factors identified as facilitators included perceptions of vaccine protection, recommendations from health professionals, positive experiences with health services and positive community attitudes towards vaccines. Our findings emphasize the need for tailored communication strategies that provide accurate and accessible information and build trust in immunization programs. Addressing structural issues such as vaccine shortages is also crucial for enhancing vaccine accessibility. By identifying both barriers and facilitators, this study provides a roadmap for designing effective interventions and policies that can increase vaccine acceptance, improve access to vaccination services and ultimately contribute to improved public health outcomes throughout Latin America.
